# The E3 ligase TRAF4 promotes IGF signaling by mediating atypical ubiquitination of IRS-1

**DOI:** 10.1016/j.jbc.2021.100739

**Published:** 2021-05-13

**Authors:** Wenjuan Yu, Ramesh Singh, Zhao Wang, Bert W. O’Malley, Ping Yi

**Affiliations:** 1 Department of Biochemistry and Molecular Biology, Baylor College of Medicine, Houston, Texas, USA; 2 Department of Molecular and Cellular Biology, Baylor College of Medicine, Houston, Texas, USA

**Keywords:** ubiquitination, insulin receptor substrate 1 (IRS-1), insulin-like growth factor (IGF), TNF receptor associated factor (TRAF), protein phosphorylation, proliferation, Akt, co-IP, coimmunoprecipitation, HA, hemagglutinin, IGF, insulin-like growth factor, IGFR, insulin-like growth factor receptor, IRS, insulin receptor substrate, PTB, phosphotyrosine binding

## Abstract

Insulin-like growth factor (IGF) is a potent mitogen that activates the IGF receptor (IGFR)/insulin receptor substrate (IRS) axis, thus stimulating growth in normal cells and uncontrolled cell proliferation in cancer. Posttranslational modifications of IRS such as ubiquitination tightly control IGF signaling, and we previously identified IRS-1 as a potential substrate for the E3 ubiquitin ligase TRAF4 using an unbiased screen. Here we provide evidence that TRAF4-mediated ubiquitination of IRS-1 is physiologically relevant and crucial for IGF signal transduction. Through site-directed mutagenesis we found that TRAF4 promotes an atypical K29-linked ubiquitination at the C-terminal end of IRS-1. Its depletion abolishes AKT and ERK phosphorylation downstream of IGF-1 and inhibits breast cancer cell proliferation. Overexpression of TRAF4 enhances IGF1-induced IGFR–IRS-1 interaction, IRS-1 tyrosine phosphorylation, and downstream effector protein activation, whereas mutation of IRS-1 ubiquitination sites completely abolishes these effects. Altogether, our studies demonstrate that nonproteolytic ubiquitination of IRS-1 is a key step in conveying IGF-1 stimulation from IGFR to IRS-1.

Insulin-like growth factors (IGFs) are important mitogenic hormones that promote cell proliferation, survival, migration, and transformation. Insulin receptor substrates (IRS) are essential cytoplasmic adaptor proteins that mediate IGF signal transduction from activated cell surface receptor (IGF-1R) to downstream effectors. IRS does not have intrinsic tyrosine kinase activity. It is phosphorylated at tyrosine residues upon binding to receptors and serves as a docking site to recruit several SH2 domain–containing proteins including PI3K, GRB-2, and SHP-2 (1). This recruitment triggers subsequent activation of AKT and ERK signaling cascades to promote downstream target gene expression.

There are six members of the IRS family. IRS-1 and IRS-2 are widely expressed in tissues and are major mediators conveying IGF and insulin signaling (2, 3). They have highly similar protein structure and both play a role in promoting primary tumor growth (4). However, they appear to have nonredundant functions.

Overexpression of IRS-1 hyperamplifies IGF signaling and contributes to tumor initiation, progression, and poor cancer prognosis (4, 5, 6). In addition to gene expression, posttranslational modification plays a pivotal role in regulating IRS functions. Tyrosine phosphorylation at the C-terminal region of IRS mediates the complex assembly of IGF-1R and different effectors. In contrast, phosphorylation at serine residues generally negatively regulates IRS function by preventing its interaction with upstream receptors or downstream effectors to limit the duration of the signal cascade (1).

IRS-1 also undergoes ubiquitination. Several E3 ubiquitin ligases, including MG53, CBL, and CUL7, have been shown to negatively regulate IRS-1 function through promoting IRS-1 protein degradation (7, 8, 9, 10, 11). Nonproteolytic ubiquitination has recently emerged as an important posttranslational modification to regulate membrane receptor-mediated signal transduction pathways (12, 13). It is not clear whether cancer cells could hijack this mechanism to regulate IRS-1 function in order to gain growth advantage. IRS-2 but not IRS-1 was reported to be modified by NEDD4 through monoubiquitination to enhance IGF-1 signaling (14). Here we report that TRAF4, a RING domain E3 ubiquitin ligase, mediates nonconventional ubiquitination of IRS-1 to promote its function.

## Results

### TRAF4 binds IRS-1 and promotes its ubiquitination

**Figure 1:**
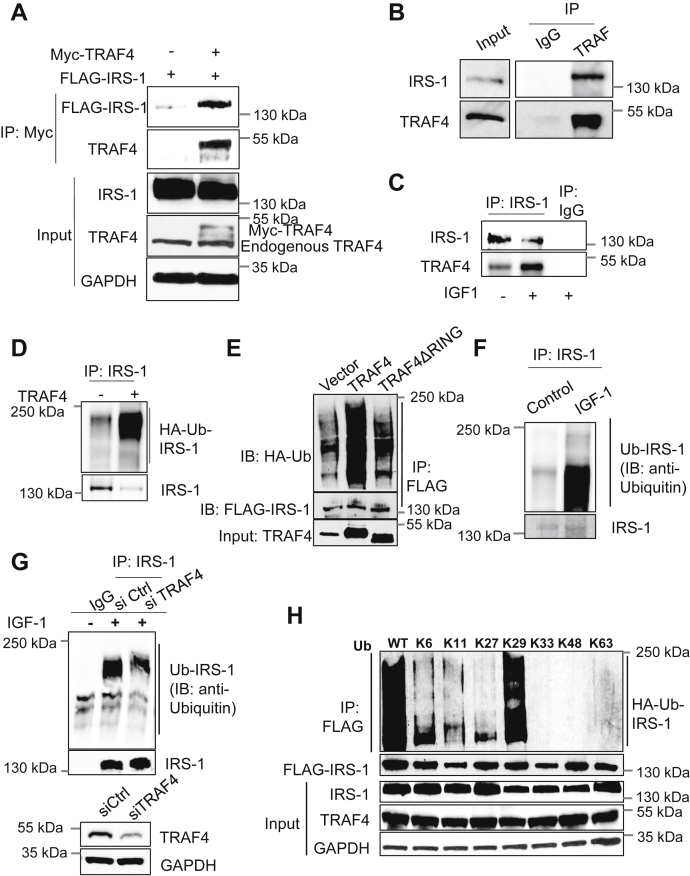
**TRAF4 promotes IRS-1 ubiquitination through K29 linkage.***A*, Myc-TRAF4 interacted with FLAG-IRS1 in transiently transfected 293T cells. A coimmunoprecipitation (co-IP) experiment was performed using an anti-Myc antibody for immunoprecipitation, followed by Western blot. *B*, endogenous IRS1 interacted with endogenous TRAF4 in MCF-7 cells. Shown is a co-IP experiment using a TRAF4-specific antibody or IgG control for immunoprecipitation. *C*, IGF-1 treatment increases the interaction between endogenous IRS-1 and TRAF4. MCF-7 cells were serum starved for 24 h followed by treatment with or without IGF-1 (100 ng/ml) for 10 min. Shown is a co-IP experiment using an IRS-1-specific antibody or IgG control for immunoprecipitation. *D*, recombinant human IRS1 protein was incubated with purified UBE1, UbcH5a, HA-tagged ubiquitin in the absence or presence of purified TRAF4 protein. An immunoprecipitation was then carried out using an IRS1-specific antibody followed by Western blot analysis using an HA-specific antibody to detect IRS-1 ubiquitination. *E*, TRAF4 but not its RING domain deletion mutant promotes IRS-1 ubiquitination. 293T cells were cotransfected with IRS-1, HA-Ub, and TRAF4 constructs as indicated. FLAG-IRS-1 was immunoprecipitated using an anti-FLAG antibody, and the ubiquitinated IRS-1 was visualized by Western blot using an anti-HA antibody. *F*, IGF-1 induced endogenous IRS-1 ubiquitination in cells. MCF-7 cells were serum starved for 24 h followed by treatment with or without IGF-1 (100 ng/ml) for 15 min. Immunoprecipitation was then performed using an anti-IRS1 antibody followed by Western blot for detection of IRS1 ubiquitination using an anti-ubiquitin antibody. *G*, TRAF4 knockdown reduced IGF-1-induced IRS-1 ubiquitination. TRAF4 was knocked down in MCF-7 cells using a mixture of control siRNAs or TRAF4 siRNAs (*top*). After 48 h, cells were serum starved for 24 h followed by treatment with or without IGF-1 (100 ng/ml) for 15 min. Immunoprecipitation was performed using an anti-IRS1 antibody followed by Western blot for detection of IRS-1 ubiquitination using an anti-ubiquitin antibody (*bottom*). *H*, TRAF4 mediates IRS-1 ubiquitination through the K29 linkage. Ubiquitination assay was performed in 293T cells after transfection with FLAG-IRS-1, TRAF4, and WT or ubiquitin mutants. K6–K63 represent the ubiquitin mutants with all lysines mutated except the indicated lysine, whereas WT indicates wildtype ubiquitin.

We previously performed an unbiased screen on a ubiquitin array to identify ubiquitination substrates for TRAF4 (15). We found that IRS-1 was among the top candidates whose ubiquitination levels significantly increased upon TRAF4 overexpression. To determine whether IRS-1 is a TRAF4-targeted ubiquitination substrate, we first evaluated the interaction between TRAF4 and IRS-1. To this end, 293T cells were transiently transfected with FLAG-tagged IRS-1 and either empty vector or myc-tagged TRAF4. Immunoprecipitation using an anti-myc antibody identified a direct association between IRS-1 and TRAF4 compared with cells expressing only FLAG-IRS-1 as a control (Fig. 1*A*). To further confirm that the TRAF4-IRS-1 interaction exists in breast cancer cells, we immunoprecipitated endogenous TRAF4 from MCF-7 cells and demonstrated a strong interaction with IRS-1 (Fig. 1*B*). A similar coimmunoprecipitation experiment using IRS-1 antibody also identified a specific association with endogenous TRAF4 (Fig. 1*C*). Furthermore, IGF-1 stimulation significantly increased the interaction between IRS-1 and TRAF4 (Fig. 1*C*). Next, we examined whether TRAF4 can promote IRS-1 ubiquitination. We performed an *in vitro* ubiquitination assay to determine whether IRS-1 is a ubiquitination substrate for TRAF4. Purified recombinant IRS-1 protein was incubated with UBE1 (E1 ubiquitin activating enzyme), UbcH5a (E2 ubiquitin conjugating enzyme), and hemagglutinin (HA)-ubiquitin, in the absence or presence of purified recombinant TRAF4 protein, for an *in vitro* ubiquitination assay. The ubiquitinated IRS-1 was detected through immunoprecipitation of IRS-1 followed by a Western blot analysis using an HA-specific antibody. As shown in Figure 1*D*, a prominent IRS-1 polyubiquitination was detected when TRAF4 was added to the *in vitro* assay, suggesting that IRS-1 is a direct TRAF4-targeted substrate. To further test whether TRAF4 mediates IRS-1 ubiquitination in cells, ubiquitination assay was performed in 293T cells transiently transfected with HA-ubiquitin, FLAG-IRS-1, and V5-TRAF4 or its ubiquitin ligase-defective RING domain deletion mutant (ΔRING). We found that wildtype TRAF4 but not the RING domain deletion mutant significantly increased the ubiquitination levels of IRS-1 in cells (Fig. 1*E*). Since IGF-1 increases the interaction of IRS-1 with TRAF4, we next examined whether endogenous IRS-1 is ubiquitinated and whether IGF-1 plays any role in it. As shown in Figure 1*F*, endogenous IRS-1 ubiquitination dramatically increased upon IGF-1 treatment. Furthermore, knockdown of TRAF4 significantly reduced IRS-1 ubiquitination (Fig. 1*G*), confirming that TRAF4 mediates IRS-1 ubiquitination upon IGF-1 stimulation.

Ubiquitin has seven lysine residues (Lys-6, Lys-11, Lys-27, Lys-29, Lys-33, Lys-48, and Lys-63) where one or more lysine residues are involved in ubiquitination chain formation. This specific ubiquitin linkage determines the fate of protein in the cell. For example, K11- and K48-linked ubiquitination is usually associated with protein degradation (16). To determine the lysine linkage involved in TRAF4-mediated IRS-1 ubiquitination, we used a series of ubiquitin mutants where all but one of the lysine residues were mutated to arginine. *In vivo* ubiquitination assay using these ubiquitin mutants identified that the K29 ubiquitin linkage is responsible for TRAF4-mediated IRS-1 ubiquitination (Fig. 1*H*). These results suggest that TRAF4 can interact with IRS-1 to promote atypical ubiquitination.

### IRS-1 or TRAF4 depletion decreased the activation of IGF-1-induced downstream effectors

**Figure 2:**
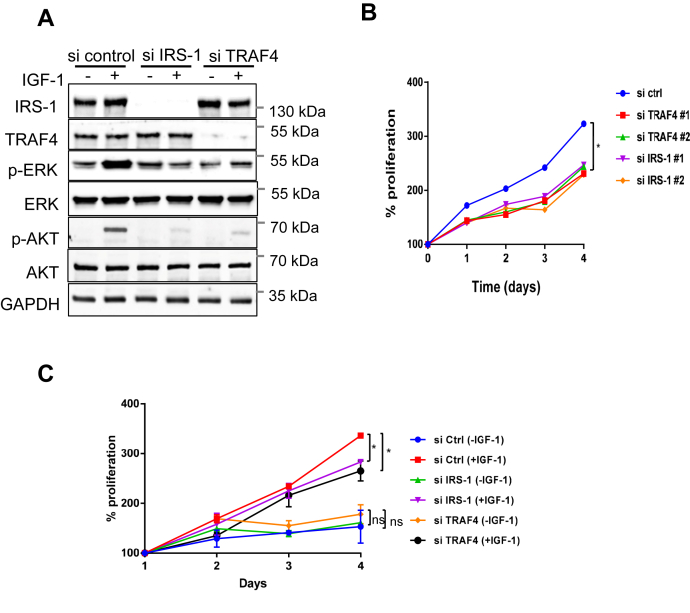
**TRAF4 regulates IRS-1-mediated IGF-1 signaling.***A*, depletion of TRAF4 or IRS-1 inhibits IGF-1-induced downstream ERK and AKT phosphorylation. IRS-1 or TRAF4 was depleted in MCF-7 cells using a pool of two different siRNAs against IRS-1 or TRAF4, respectively. MCF-7 cells were then serum starved for 24 h and treated with or without IGF-1 (100 ng/ml) for 15 min. Gene knockdown efficiency and ERK and AKT phosphorylation were determined by Western blot. *B*, depletion of TRAF4 or IRS-1 inhibits MCF-7 cell proliferation. TRAF4 or IRS-1 was knocked down using two different TRAF4 or IRS-1 siRNAs, respectively, or transfected with control siRNA in MCF-7 cells. Cell proliferation was determined for indicated period of time using MTS assay. ∗*p* < 0.05 by two-way ANOVA with Tukey’s multiple comparisons test. Data are presented as mean ± SD. *C*, IRS-1 or TRAF4 depletion reduced IGF-1-induced cell proliferation. These MCF-7 cells transfected with control, IRS-1 or TRAF4 siRNA were serum starved and treated with or without IGF-1 followed by cell proliferation assay. Cell proliferation was determined for indicated periods using an MTS assay. ∗*p* < 0.05 by two-way ANOVA with Tukey’s multiple comparisons test. Data are presented as mean ± SD.

To determine whether TRAF4 affects the IGF-1-induced signaling cascade, we first analyzed the effect of IRS-1 or TRAF4 knockdown in MCF-7 cells on the activation of proteins in the MAPK signaling pathway and PI3K-AKT signaling pathway, which are known downstream effectors of IRS-1. IRS-1 or TRAF4 was depleted in MCF-7 cells using a pool of two different siRNAs against IRS-1 or TRAF4, respectively. The phosphorylation levels of ERK and AKT significantly increased upon IGF-1 stimulation for 15 min, whereas the total protein levels remain unchanged (Fig. 2*A*), an indication of IGF-1-induced ERK and AKT activation. Knockdown of IRS-1 or TRAF4 significantly reduced IGF-1-induced ERK and AKT phosphorylation. The protein levels of total ERK and AKT were not decreased upon IRS-1 or TRAF4 knockdown (Fig. 2*A*). These results indicate that knockdown of TRAF4 presents the same effects as the IRS-1 and abolishes IGF-1-induced downstream pathway activation. Furthermore, the total protein level of IRS-1 remained unchanged upon TRAF4 knockdown with or without IGF-1 stimulation, indicating a unique role of TRAF4 in IGF signal regulation other than inducing proteasomal degradation of ubiquitinated IRS-1 to suppress IGF signaling.

Since IRS-1 promotes cell proliferation (2), we next examined whether TRAF4 also plays a role in regulating cell growth. To test this, TRAF4 was knocked down using two different siRNAs in MCF-7 cells. We also knocked down the IRS-1 using two different siRNAs in MCF-7 cells to compare its effect on cell proliferation with TRAF4 knockdown. The cell growth rate was monitored using MTS assay. We found that either TRAF4 or IRS-1 depletion significantly inhibited MCF-7 cell proliferation as compared with control siRNA (Fig. 2*B*). We also examined the effects of TRAF4 or IRS-1 knockdown on cell proliferation in the absence or presence of IGF-1 stimulation. As shown in Figure 2*C*, TRAF4 or IRS-1 depletion reduced MCF-7 cell proliferation in the presence of IGF-1. Both have minor effects in the absence of IGF-1. These results suggest that TRAF4, similar to IRS-1, plays a role in IGF-1-stimulated MCF-7 cell proliferation.

### Overexpression or knockdown of TRAF4 affects IRS-1 tyrosine phosphorylation through regulating the interaction between IRS-1 and IGF-1R

**Figure 3:**
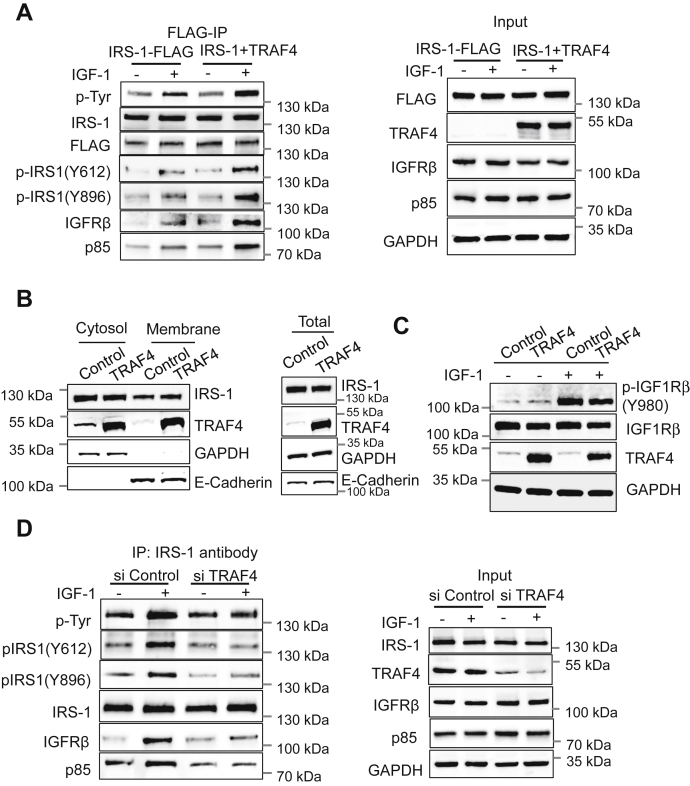
**TRAF4 regulates the interaction of IRS-1 with IGFR and IRS-1 tyrosine phosphorylation upon IGF-1 stimulation.***A*, TRAF4 overexpression increased IGF-1-induced IRS-1-IGFR interaction and IRS-1 tyrosine phosphorylation. MCF-7 cells were transfected with FLAG-IRS-1 with and without TRAF4, serum starved for 24 h, and then treated with or without IGF-1 (100 ng/ml) for 15 min. Immunoprecipitation was performed using an anti-FLAG antibody followed by Western blot (*left*). TRAF4 and FLAG-IRS-1 overexpression and endogenous expression of indicated proteins were determined by Western blot. GAPDH was used as internal control (*right*). *B*, TRAF4 does not alter IRS-1 membrane recruitment in the presence of IGF-1. MCF-7 cells were transfected with vector control or TRAF4 expression plasmid, serum starved, and then stimulated with IGF-1 for 10 min before harvesting. Cytosol and membrane fractions were prepared using a Mem-PER plus membrane protein extraction kit. Western blot was performed to determine the distribution of IRS-1 in cytosol and membrane fraction using anti-IRS1 antibody. GAPDH was used as loading control for cytosol and whole-cell lysate fraction, whereas E-cadherin was used as loading control for the membrane fraction. *C*, TRAF4 does not alter the protein level and the phosphorylation level of IGF-1Rβ. MCF-7 cells were transfected with either vector control or TRAF4 plasmids. These cells were serum starved for 24 h followed by treatment with or without IGF-1 (100 ng/ml) for 5 min. Cell lysate was prepared followed by Western blot to detect phosphorylation levels of IGF-1Rβ. *D*, deletion of TRAF4 abolished IGF-1-induced interaction between IRS-1 and IGFR. MCF-7 cells were transfected with a pool of two different siRNAs against TRAF4 or control siRNA. MCF-7 cells were then serum starved for 24 h followed by treatment with or without IGF-1 (100 ng/ml) for 15 min. Immunoprecipitation was performed using an anti-IRS-1 antibody followed by Western blot (*left*). TRAF4 knockdown efficiency and endogenous expression of indicated proteins were determined by Western blot. GAPDH was used as internal control (*right*).

Next, we investigated the underlying mechanisms by which TRAF4 affects the IGF-1-induced signal transduction cascade. IGF binds to the IGF receptor (IGF-1R) in the plasma membrane and induces the activation of its intrinsic tyrosine kinase and autophosphorylation. The activated IGF-1R recruits and phosphorylates the intracellular substrate IRS-1, and the phosphorylated IRS-1 is in turn recognized by SH2 domain–containing proteins, such as the p85 regulatory subunit of PI3K, and triggers activation of downstream signaling pathways. Since we found that TRAF4 affected the activation of IGF-1-induced downstream effectors, we first analyzed the levels of total tyrosine phosphorylation of IRS-1 in the absence or presence of TRAF4 overexpression. We found that TRAF4 overexpression increased the total phosphorylated tyrosine level of IRS-1 compared with that in the absence of TRAF4 overexpression upon IGF-1 stimulation (Fig. 3*A*). In the absence of IGF-1 stimulation, TRAF4 overexpression did not alter the levels of IRS-1 tyrosine phosphorylation, suggesting that TRAF4 specifically affects an IGF-1-induced signaling event(s). The phosphorylation of many tyrosine residues of IRS-1 is involved in the recruitment of downstream proteins, of which Y612 is one of the most crucial PI3K-binding tyrosine residues and Y896 is the GRB2-binding tyrosine. We next examined the levels of IRS-1 phosphorylation at Y612 and Y896. Phosphorylation levels of these residues were elevated in the presence of TRAF4 overexpression compared with control upon IGF-1 stimulation (Fig. 3*A*).

IRS-1 is recruited to cell membrane to interact with IGF-1R upon IGF-1 stimulation. It was reported previously that NEDD4-mediated IRS-2 ubiquitination recruits IRS-2 to the membrane (14). To determine whether TRAF4-mediated ubiquitination affects IRS-1 membrane localization, we separated the membrane and cytoplasmic fractions of lysates from IGF-1-treated MCF-7 cells with or without TRAF4 overexpression (Fig. 3*B*). A significant portion of TRAF4 was found to be associated with membrane. However, the levels of membrane-associated IRS-1 was not significantly different comparing cells with or without TRAF4 overexpression, suggesting that TRAF4-mediated IRS-1 ubiquitination does not play a role in IRS-1 membrane recruitment. Since activated IGF-1R phosphorylates IRS-1, we first examined whether TRAF4 affects IGF-1-induced IGF-1R activation in control or TRAF4-overexpressing MCF-7 cells. The Y980 phosphorylation level of IGF-1R dramatically increased in the presence of IGF-1, indicating an active status of the receptor (Fig. 3*C*). However, TRAF4 overexpression did not affect the level of IGF-1R phosphorylation. Next, we investigated whether TRAF4 regulates IGF-1-induced IRS-1 recruitment to IGF-1R. An increased association of IGF-1Rβ with IRS-1 was observed following IGF-1 stimulation. TRAF4 overexpression further augmented this interaction (Fig. 3*A*), indicating that TRAF4 enhances IGF-1-induced tyrosine phosphorylation of IRS-1 through promoting the interaction between IRS-1 and IGF-1R. The amount of p85 bound to IRS-1 upon IGF-1 stimulation also was significantly increased when TRAF4 is overexpressed, consistent with the level of IRS-1 tyrosine phosphorylation. We further knocked down endogenous TRAF4 in MCF-7 cells and found that TRAF4 depletion abolished IGF-1-induced interaction between IGF-1Rβ and IRS-1; the upregulation of IRS-1 tyrosine phosphorylation, including Y612 and Y896 phosphorylation; and the induced association between p85 and IRS-1 compared with the nontargeting control (Fig. 3*D*). These results suggest that TRAF4 is an essential protein in mediating IGF-1 signal transduction.

### IRS-1 C-terminal lysine residues are responsible for TRAF4-mediated ubiquitination

**Figure 4:**
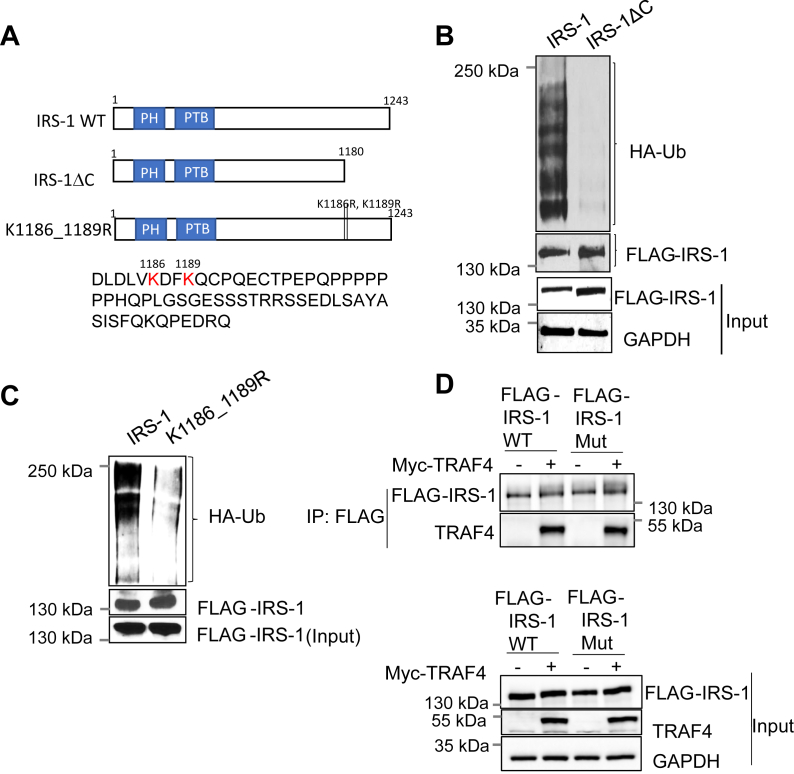
**TRAF4 targets K1186 and K1189 of IRS-1 for ubiquitination.***A*, schematic representation of IRS-1 WT and the mutants. The C-terminal last 62 amino acid sequences are shown with K1186 and K1189 in *red letter*. *B*, deletion of the C-terminal (1181–1243) region of IRS-1 abolished TRAF4-mediated ubiquitination. FLAG-IRS-1 and deletion mutant was cotransfected with TRAF4 and HA-Ub into 293T cells. The IRS-1 was immunoprecipitated using a FLAG antibody, and the ubiquitinated IRS-1 was detected using an anti-HA antibody by Western blot. *C*, mutation of lysine 1186 and 1189 to arginine in IRS-1 abolished TRAF4-mediated ubiquitination. FLAG-IRS-1 WT or K1186_1189R mutant was cotransfected with TRAF4 and HA-Ub into 293T cells. The IRS-1 was immunoprecipitated using a FLAG antibody, and ubiquitinated IRS-1 was detected using an anti-HA antibody by Western blot. *D*, IRS-1 ubiquitination site mutation does not abolish the interaction between IRS-1 and TRAF4. *Top*, Myc-TRAF4 interacted with FLAG-IRS-1 WT or TRAF4-ubiquitin mutant IRS-1 (IRS-1 mut) in transiently transfected 293T cells. A coimmunoprecipitation experiment was performed using an anti-FLAG antibody for immunoprecipitation, followed by Western blot. *Bottom*, input control.

IRS-1 is a 185-kDa phosphoprotein that consists of a pleckstrin homology domain and a phosphotyrosine binding (PTB) domain near the N-terminal region. The center and the C terminus of the IRS-1 proteins contain several tyrosine-phosphorylation sites that bind to intracellular molecules that contain SH2 domains (1, 3). Posttranslational modifications in the N- or C-terminal regions of IRS-1 have been shown to have a positive impact on insulin signaling pathways (17). To identify potential TRAF4-targeted ubiquitination sites, we generated a deletion mutant of IRS-1 (Fig. 4*A*) and found that deletion of the 62 amino acids from the IRS-1 C-terminal end (IRS-1ΔC) abolished TRAF4-mediated IRS-1 ubiquitination (Fig. 4*B*). These results suggest that this IRS-1 deletion region is likely the target of TRAF4-mediated ubiquitination. Ubiquitin ligases often target multiple neighboring lysine residues for ubiquitination. There are three lysine residues present in the IRS-1 deletion domain, where two of them (K1186 and K1189) are located close to each other (Fig. 4*A*). To test whether these two lysine residues are TRAF4-targeted ubiquitination sites, we used site-directed mutagenesis to generate lysine to arginine mutants of IRS-1. Mutation of two of these lysine residues to arginine (K1186R and K1189R) significantly reduced TRAF4-mediated ubiquitination (Fig. 4*C*). These results suggest that TRAF4 targets IRS-1 ubiquitination at the K1186 and K1189 residues. We found that the IRS-1 mutant is still able to bind TRAF4 (Fig. 4*D*), suggesting that the interaction between TRAF4 and IRS-1 does not depend on TRAF4-targeted ubiquitination sites.

### TRAF4-mediated IRS-1 ubiquitination regulates IRS-1 tyrosine phosphorylation and its interaction with IGF-1R

**Figure 5:**
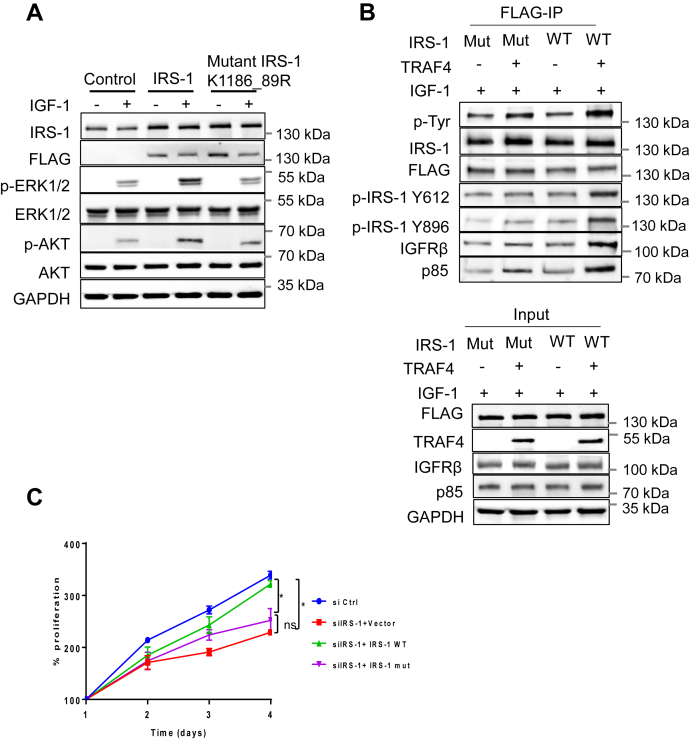
**Mutation of TRAF4-targeted IRS-1 ubiquitination sites inhibits IGF-1 signaling and TRAF4-promoted IRS-1 tyrosine phosphorylation.***A*, IRS-1 ubiquitination mutant failed to induce downstream ERK and AKT phosphorylation upon IGF-1 stimulation. MCF-7 cells were transfected either with control vector, FLAG-IRS-1 WT or mutant IRS-1 (K1186_1189R). MCF-7 cells were then serum starved for 24 h and treated with or without IGF-1 (100 ng/ml) for 15 min. Western blot was performed to determine the level of ERK and AKT phosphorylation. *B*, mutation of K1186 and K1189 of IRS-1 inhibits TRAF4-promoted IRS-1 tyrosine phosphorylation and its interaction with IGFR. 293T cells were cotransfected with FLAG-tagged wildtype or mutant IRS-1 in the absence or presence of TRAF4. The cells were then serum starved for 24 h and treated with IGF-1 (100 ng/ml) for 15 min. The wildtype or mutant IRS-1 was then immunoprecipitated using an anti-FLAG antibody. Western blot was then performed to determine IRS-1 phosphorylation level and its interaction with IGFR (*top*). Overexpression of TRAF4, IRS-1 WT, and mutant was determined by Western blot. GAPDH was used as an internal control (*bottom*). *C*, IRS-1 wildtype but not the ubiquitination mutant rescued the effect of siIRS-1 on cell proliferation. Shown is a cell proliferation assay in MCF-7 cells after IRS-1 knockdown and transfection with either WT IRS-1 or IRS-1 mutant. ∗*p* < 0.05 by two-way ANOVA with Tukey’s multiple comparisons test. Data are presented as mean ± SD.

To elucidate whether TRAF4-mediated IRS-1 ubiquitination is required for TRAF4-induced activation of IGF-1 downstream effectors, we first examined the effects of IRS-1 or mutant IRS-1 K1186_89R overexpression in MCF-7 cells on the IGF-1-induced activation in MAPK and PI3K-AKT signaling pathways. The wildtype and mutant IRS-1 have comparable expression levels in transfected MCF-7 cells since IRS-1 levels were elevated to a similar level in the two cells compared with those in the control cells (Fig. 5*A*). Transfection of wildtype IRS-1 significantly increased the levels of p-ERK and p-AKT in the presence of IGF-1. Transfection of the mutant IRS-1 K1186_89R, however, did not have any effect. These results suggest that the IRS-1 ubiquitination-defective mutant lost the ability to activate downstream signaling.

**Figure 6:**
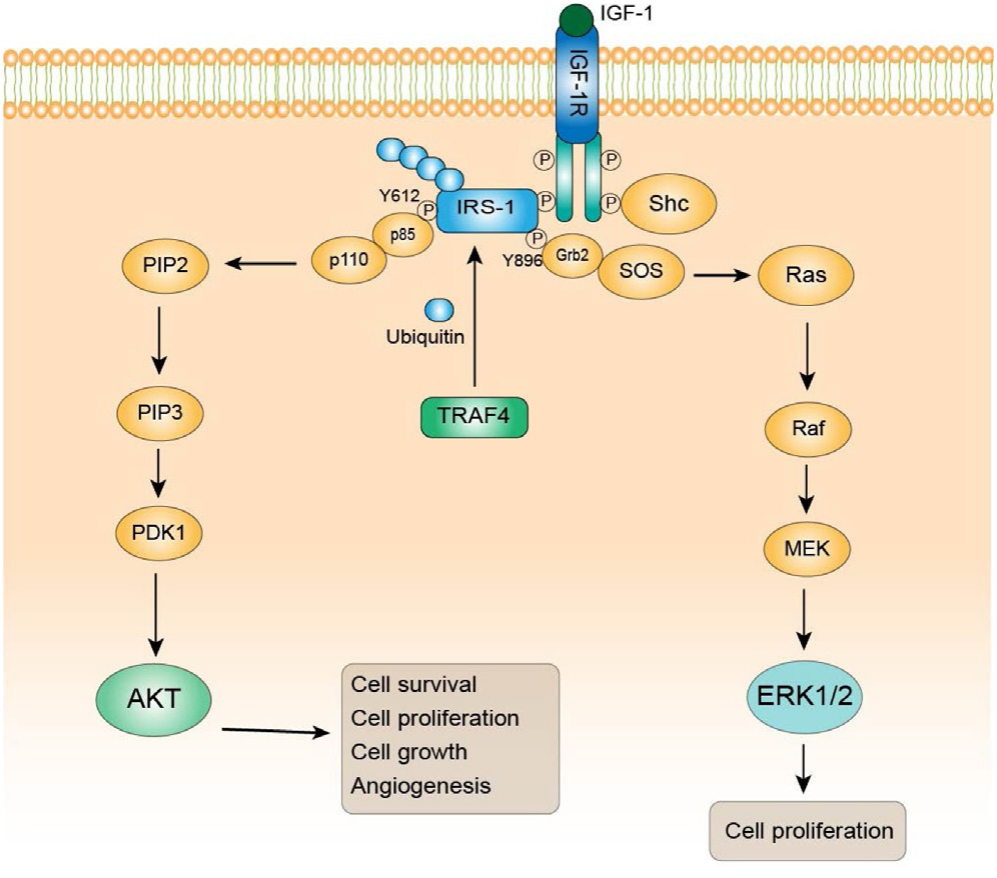
**Working model for regulation of IGF-1 signaling by TRAF4-mediated IRS-1 ubiquitination in breast cancer cell line.** TRAF4 overexpression in cancer cell leads to atypical K-29 linked IRS-1 ubiquitination at the lysine position 1186 and 1189 at the C-terminal domain. IRS-1 ubiquitination promotes its interaction with IGFR that leads to increased IRS-1 tyrosine phosphorylation (Y612, Y896). IRS-1 is an adaptor protein, and increased IRS-1 phosphorylation facilitates the recruitment of downstream effectors such as p85 and Grb2, resulting in enhanced ERK and AKT phosphorylation and hyperactivated IGF-1 signaling cascade. Consequently, increased IGF signaling promotes cell proliferation, survival, and growth.

We further tested whether IRS-1 ubiquitination is required for TRAF4 to regulate IRS-1 function. To this end, we transiently transfected FLAG-tagged wildtype or mutant IRS-1 in the absence or presence of TRAF4 cotransfection into 293T cells. The wildtype or mutant IRS-1 was then immunoprecipitated using an anti-FLAG antibody. We found that TRAF4 overexpression failed to enhance mutant IRS-1 total tyrosine phosphorylation, Y612 and Y896 phosphorylation in contrast to its effects on wildtype IRS-1 (Fig. 5*B*). The levels of IGF-1Rβ and p85 bound to IRS-1 also are not elevated in the IRS-1 mutant cells (Fig. 5*B*). These results suggest that IRS-1 tyrosine phosphorylation is impaired by the mutation of the IRS-1 ubiquitination site, indicating that the ubiquitination at IRS-1(K1186_89) is essential for TRAF4-induced enhancement of IRS-1 tyrosine phosphorylation. Since TRAF4 or IRS-1 depletion inhibits cell proliferation, we next examined the ability of IRS-1 WT or the ubiquitination mutant to rescue the effect of IRS-1 depletion on cell proliferation. We found indeed IRS-1 WT but not the mutant restored the cell proliferation (Fig. 5*C*). Altogether, our results demonstrate that TRAF4-mediated IRS-1 ubiquitination leads to the enhancement of IRS-1 tyrosine phosphorylation through promoting the interaction between IRS-1 and IGF-1R; the phosphorylated IRS-1 in turn binds p85 regulatory subunit of PI3K to activate the downstream effectors (Fig. 6).

## Discussion

IRS-1 is a key adaptor protein in IGF-1 signaling transduction. In the present study, we examined the regulation of IRS-1 by TRAF4-mediated nonproteolytic ubiquitination and its effect on IGF-1R signaling. TRAF4 is an E3 ubiquitin ligase that is overexpressed in breast carcinoma (18). Herein we demonstrated that TRAF4-mediated IRS-1 ubiquitination regulates IRS-1 binding with IGF-1R and subsequent IRS-1 phosphorylation. These events lead to activation of both MAPK and PI3K signaling pathways that stimulates proliferation in the MCF-7 breast cancer cell line.

IRS-1 has been associated with breast cancer progression with poor differentiation, cell survival, proliferation, and motility. It is reported to be constitutively activated in breast cancer tumors (19, 20). High levels of IRS-1 associate with lower disease-free survival and positively correlate with proliferation in estrogen receptor–positive breast cancer tumors (6, 21). IRS-1 silencing also promotes apoptosis and increases the sensibility to chemotherapy in estrogen receptor–positive breast cancer cells (22). IRS-1 has been shown to bind not only to IR and IGF-1R but also to several other proteins. It has been reported that the rapamycin-dependent pathway acts as a negative regulator of IRS-1 by increasing its serine/threonine phosphorylation, followed by its degradation (23). Various E3 ubiquitin ligases promote IRS-1 protein degradation including MG53, CBL, and CUL7 (7, 8, 9, 10). We demonstrate here that IRS-1 also undergoes nonproteolytic ubiquitination. It is a substrate for TRAF4-mediated ubiquitination (Fig. 1, *D* and *E*) through atypical K29-linked ubiquitin chains (Fig. 1*H*).

The well-studied K48 and K63 polyubiquitin chain mainly leads to protein degradation. However, the functional role of K29 ubiquitin linkages has not been studied extensively. It was reported to disrupt protein–protein interaction (24), disassemble protein complexes (25), and regulate protein localization and migration (26). We found that IRS-1 is ubiquitinated at the K1186 and K1189 residues by TRAF4 (Fig. 4*C*). These lysine residues are located adjacent to each other in the C-terminal domain, which is also the effector binding site. This region contains multiple tyrosine phosphorylation sites, which during IGF stimulation are phosphorylated and act as docking sites for GRB2, SHP2, and PI3K (1). The two ubiquitination sites are particularly close to SHP2 docking sites at Y1172 and Y1222 (27). SHP2 is a tyrosine phosphatase that is activated upon binding to IRS-1 (28). It dephosphorylates IRS-1 and attenuates insulin signaling pathways (27). It is possible that the polyubiquitin chain at the K1186 and K1189 sites interfere with SHP2 recruitment, resulting in highly phosphorylated IRS-1 and strong activation of downstream signaling, similar to the effects observed for SHP2 docking-site mutations (27). Another possibility could be that the K29-linked ubiquitin chain conjugated at the K1186 and K1189 sites stabilizes the binding of IRS-1 and IGF-1R. Although the ubiquitination sites are located at the C-terminal end of IRS-1 while its interaction with IGF-1R occurs at the N-terminal PTB domain (29), the spatial distance between the two regions could be close. The long polyubiquitin chain might serve as a bridge to further stabilize the interaction between the IRS-1 PTB domain and the phosphorylated NEPY motif within the juxtamembrane region of IGF-1R (29). In line with this hypothesis, we found that TRAF4-mediated IRS-1 ubiquitination indeed leads to an increased IRS-1/IGF-1R interaction (Fig. 3*A*). This increased interaction was abolished when these lysine residues were mutated (Fig. 5*B*).

Among all IRSs, IRS-1 and IRS-2 are ubiquitously expressed with high expression in various cancer cell lines (1, 30). Several reports clearly demonstrate the nonredundant functions of IRS-1 and IRS-2. IRS-1 is responsible for tumor proliferation, whereas IRS-2 promotes cancer cell motility and invasion (4, 30). Of interest, TRAF4-mediated ubiquitination sites for IRS-1 (K1186 and K1189) are not conserved in IRS-2. It was reported that Nedd4 promotes IRS-2 but not IRS-1 ubiquitination and the IRS-2 ubiquitination site is not conserved in IRS-1 (14). It is likely that TRAF4 and Nedd4 differentially regulate IRS-1 and IRS-2 ubiquitination and subsequent IGF-1 signaling in cancer cells.

IGF-1 binds and activates the tyrosine kinase receptor IGF-1R, leading to multisite autophosphorylation of the IGF-1R β-subunit, tyrosine phosphorylation, and recruitment of cellular substrates IRS-1 and SHC proteins, which in turn act as docking sites for other proteins (31, 32). Phosphorylation of IRS-1 at Y612 acts as a binding site for the p85 regulatory subunit of PI3K *via* its Src homology 2 domains, whereas Y896 acts as a Grb2-binding site. We found that knockdown of IRS-1 leads to reduced phosphorylation of AKT and ERK upon IGF-1 treatment in MCF-7 cells. Of interest, knockdown of TRAF4 in MCF-7 cells had a similar outcome with reduced AKT and ERK phosphorylation under similar conditions possibly indicating a more general role for TRAF4 in IRS-1 mediated IGF-1R signaling (Fig. 2*A*). Furthermore, we examined IRS-1 phosphorylation at Y612 and Y896, which is responsible for recruitment of p85 and Grb2, respectively. We observed that TRAF4 overexpression leads to increased IRS-1 phosphorylation at Y612 and Y896 (Fig. 3*A*). There also was increased IGF-1R binding and recruitment of the PI3K subunit, p85, with TRAF4 overexpression. With the IRS-1 ubiquitin mutant we found reduced AKT and ERK phosphorylation upon IGF-1 treatment. IRS-1 ubiquitin mutant also had reduced phosphorylation at Y612 and Y896 with TRAF4 overexpression. There was a diminished interaction or binding between the IRS-1 ubiquitin mutant and IGF-1R and p85 with TRAF4 overexpression (Fig. 5*B*). All these results support the notion that TRAF4-mediated IRS-1 ubiquitination regulates IRS-1 phosphorylation and subsequent IGF-1R signaling and AKT/ERK pathway activation (Fig. 6).

We also investigated the effect of TRAF4 on MCF-7 cell proliferation since TRAF4-mediated IRS-1 ubiquitination leads to AKT and ERK activation. Activation of AKT and ERK signaling increases proliferation in cells. Cell proliferation assay in MCF-7 cells indicates that knockdown of TRAF4 using siRNA leads to a significant decrease in cell proliferation activity. This effect was similar to the knockdown effects of IRS-1 in MCF-7 cells (Fig. 2*B*). This further substantiates the role of TRAF4 in IRS-1-mediated insulin signaling and cell proliferation.

Although the IRS-1 protein lacks intrinsic kinase activity, it serves as a scaffold to initiate intracellular signaling pathways. Since IRS-1 is a crucial intermediate for multiple receptors and signaling pathways that can influence tumor progression, it is a pivotal target for therapeutic intervention.

Taken together, our study establishes a novel role of TRAF4 in the regulation of IGF-1R signaling through nonproteolytic ubiquitination of IRS-1. Since IGF signaling plays a very important role in various pathological conditions including cancer, regulation of this pathway by TRAF4 presents a potential biomarker and a therapeutic target for intervention.

## Experimental procedures

### Cell culture

The human breast cancer cell line MCF7 and human embryonic kidney epithelial cell line HEK293T were obtained from American Type Culture Collection. MCF7 cells were maintained in Dulbecco's modified Eagle's medium containing 10% fetal bovine serum, 2 mM L-glutamine, 100 μg/ml streptomycin, and 100 U/ml penicillin at 37 °C and 5% CO₂. HEK293T cells were maintained in Dulbecco's modified Eagle's medium supplemented with 10% fetal bovine serum, 100 μg/ml streptomycin, and 100 U/ml penicillin at 37 °C and 5% CO₂. For human insulin-like growth factor-1 (IGF-1) treatment experiments, cells were maintained in serum-free culture medium for 24 h supplemented with 2 mM L-glutamine, 100 U/ml penicillin, and 100 μg/ml streptomycin and then treated with IGF-1 (100 ng/ml) for 15 min unless otherwise indicated.

### Reagents and antibodies

Human IGF-1 (catalog 13769) was obtained from Sigma-Aldrich. Primary antibodies used were as follows: anti-Phospho-Akt (Ser473) (catalog 4060), anti-Akt (catalog 9272), anti-Phospho-p44/42 MAPK (Erk1/2) (Thr202/Tyr204) (catalog 4377), anti-p44/42 MAPK (Erk1/2) (catalog 9102), anti-PI3 Kinase p85(catalog 4292) and anti-IGF-1 Receptor β-subunit (D23H3) (IGF-1R β, catalog 9750) from Cell Signaling Technology; anti-TRAF4 (catalog ab108991) and anti-IRS-1 (Phospho Y896, catalog ab 4873) from Abcam; anti-Ubiquitin (catalog sc-8017) and anti-GAPDH (catalog sc-32233) from Santa Cruz Biotechnology Inc; anti-IRS-1 (21HCLC) (catalog 71009) and anti-Phospho-IRS-1(Y612) (catalog 44-816G) from Thermo Fisher Scientific; anti-Phosphotyrosine (pTyr, clone 4G10, catalog 05-321) from Sigma-Aldrich. HRP-conjugated secondary anti-mouse (catalog 1706516) and anti-rabbit (catalog 1706515) antibodies were obtained from Bio-Rad. Monoclonal ANTI-FLAG M2-peroxidase (HRP) antibody (catalog 8592A) and EZview Red ANTI-FLAG M2 Affinity Gel (catalog F2426) were obtained from Sigma-Aldrich. Protein A-Agarose beads (catalog sc-2001) were obtained from Santa Cruz Biotechnology. Recombinant human IRS-1 protein (catalog ab70538) was obtained from Abcam. This truncated recombinant protein has amino acids from 600 to 1245, which includes TRAF4-targeted lysines for ubiquitination.

### Construction of expression vectors and IRS-1 mutants and transfection

The *TRAF4* cDNA was cloned into pSG5 expression vector without FLAG-tag. All *IRS-1* and *IRS-1* deletion as well as lysine mutants were cloned into FLAG-tagged pSG5 expression vector using pBS human *IRS-1* (Addgene plasmid # 11359; http://n2t.net/addgene:11359; RRID:Addgene_11359) as a PCR template. Cells were transfected with plasmid DNA using Lipofectamine 3000 (Thermo Fisher Scientific) following the manufacturer’s protocol.

### siRNA and transfection

The siRNA (Silencer Select) for IRS-1 #1 (ID s7521), IRS-1 #2 (ID s7522), TRAF4 #1 (ID s18479), TRAF4 #1 (s225169), and negative control siRNA (catalog 4457289) were obtained from Thermo Fisher Scientific and transfected into MCF7 cells using Lipofectamine RNAiMAX transfection reagent (Thermo Fisher Scientific) as per manufacturer’s protocol.

### Cytosol and membrane protein extraction

The cytosol and membrane protein fractions were isolated from the cells using Mem-PER plus membrane protein extraction kit (Catalog 89842, Thermo Fisher Scientific) as per manufacturers protocol. Briefly, cells were collected in the growth medium followed by centrifugation at 300*g* for 5 min. The cell pellet was washed twice in cell wash solution followed by centrifugation. Thereafter, the cell pellet was incubated in permeabilization buffer for 10 min at 4 °C with constant mixing followed by centrifugation for 15 min at 16,000*g*. The supernatant obtained is the cytosolic fraction. The residual cell pellet was then solubilized in solubilization buffer at 4 °C for 30 min with constant mixing. The tubes were then centrifuged at 16,000*g* for 15 min at 4 °C to collect the membrane protein fraction. The cytosolic and membrane fractions were stored at −80 °C for further use.

### Western blot

Cells were harvested, and protein was extracted from the cells using NP40 cell lysis buffer (catalog FNN0021, Thermo Fisher Scientific) supplemented with 1 mM DTT, 1X protease inhibitor cocktail (catalog p8340, Sigma-Aldrich). The protein concentration was determined using a protein assay kit (Bio-Rad), and samples were separated in SDS polyacrylamide gels with various concentrations depending on the molecular weight of the protein studied. Proteins were then transferred onto a polyvinylidene difluoride membrane. After probing with a primary antibody at 4 °C overnight, the membrane was incubated with a secondary antibody conjugated with HRP. Finally, the signal intensity was determined using Pierce ECL Western Blotting Substrate (catalog 32109, Thermo Fisher Scientific) and Azure imaging system c600 (Azure Biosystems, Inc). Endogenous GAPDH was used as the internal control.

### *In vitro* ubiquitination assay

His and FLAG-tagged recombinant human TRAF4 was purified from Sf9 cells infected with TRAF4-expressing baculovirus (produced in BCM Monoclonal Antibody/recombinant Protein Expression Core Facility) through Nickle and FLAG affinity purifications. Recombinant human IRS-1 protein (Abcam) was incubated with 100 ng UBE1, 150 ng UBE2D1 (UbcH5a), and 5 μg HA-ubiquitin (Boston Biochem) in the absence or presence of 500 ng purified recombinant TRAF4 with ubiquitination buffer (50 mM Tris-Cl, pH 7.4, 2 mM ATP, 5 mM MgCl2, 2 mM DTT) at 30 °C for 90 min. The incubation mixture was then subjected to immunoprecipitation using an anti-IRS1 antibody, followed by Western blot analysis using an anti-HA antibody.

### Immunoprecipitation

Cell lysis was performed using cold NP40 cell lysis buffer supplemented with 1 mM DTT, 1X protease inhibitor cocktail and 1 × Halt phosphatase inhibitor single-use cocktail (catalog 78428, Thermo Fisher Scientific). The lysates were then centrifuged at 15,000*g* for 10 min at 4 °C, and the protein concentration was determined using a protein assay kit (Bio-Rad). For immunoprecipitation, equal amounts of proteins of each sample were mixed with indicated antibodies. FLAG-tagged proteins were immunoprecipitated using EZview Red ANTI- FLAG M2 affinity gels. For the immunoprecipitation of endogenous IRS-1, protein A-Agarose beads and anti-IRS-1 antibody were used. Samples were incubated on rotor at 4 °C for 3 h. After thoroughly washing the beads with wash buffer (1% NP40, 1 mM DTT, 1 × protease inhibitor) three times, an equal volume of 2X Laemmli buffer (0.4 M Tris-HCl pH 6.8, 8% SDS, 20% glycerol, 10% 2-mercaptoethanol, 0.2% bromophenol blue) was added before boiling the samples for 5 min. The immune complexes were analyzed by Western blot analyses using specific antibody.

### Cell proliferation assay

The cell proliferation assay was performed using CellTiter 96 AQueous One Solution Cell Proliferation Assay (MTS) reagent (catalog G358A, Promega) according to the manufacturer’s instructions. Briefly, MCF-7 cells were seeded in a 96-well plate 48 h after TRAF4 or IRS-1 knockdown using two different siRNAs or control siRNA. The plate was incubated at 37 °C in a humidified, 5% CO_2_ atmosphere for 4 days. For daily reading, 20 μl CellTiter 96 AQueous One Solution Reagent was added to specific wells containing 100 μl media and incubated for 3 h. Absorbance was measured at 490 nm using a microplate reader. Cell proliferation of individual samples was calculated by normalizing their absorbance to that of the corresponding control sample.

### Statistics

Unless otherwise indicated, all results represent mean ± SD, and statistical comparisons between different groups were performed using two-way ANOVA with multiple comparisons corrections. For all statistical analyses, differences of *p* < 0.05 were considered statistically significant. GraphPad Prism software version 7.0 (GraphPad Software) was used for data analysis.

## Data availability

All data are contained within the article.

## Conflict of interest

The authors declare that they have no conflicts of interest with the contents of this article.
